# Dynamic Contextual Modulation in Superior Colliculus of Awake Mouse

**DOI:** 10.1523/ENEURO.0131-20.2020

**Published:** 2020-09-15

**Authors:** Gioia De Franceschi, Samuel G. Solomon

**Affiliations:** Institute of Behavioural Neuroscience, Department of Experimental Psychology, University College London, London WC1H 0AP, United Kingdom

**Keywords:** adaptation, functional properties, non-classical receptive field, suppression, tectum, vision

## Abstract

The responses of neurons in the visual pathway depend on the context in which a stimulus is presented. Responses to predictable stimuli are usually suppressed, highlighting responses to unexpected stimuli that might be important for behavior. Here, we established how context modulates the response of neurons in the superior colliculus (SC), a region important in orienting toward or away from visual stimuli. We made extracellular recordings from single units in the superficial layers of SC in awake mice. We found strong suppression of visual response by spatial context (surround suppression) and temporal context (adaptation). Neurons showing stronger surround suppression also showed stronger adaptation effects. In neurons where it was present, surround suppression was dynamic and was reduced by adaptation. Adaptation’s effects further revealed two components to surround suppression: one component that was weakly tuned for orientation and adaptable, and another component that was more strongly tuned but less adaptable. The selectivity of the tuned component was flexible, such that suppression was stronger when the stimulus over the surround matched that over the receptive field. Our results therefore reveal strong interactions between spatial and temporal context in regulating the flow of signals through mouse SC, and suggest the presence of a subpopulation of neurons that might signal novelty in either space or time.

## Visual Abstract

**Figure:**
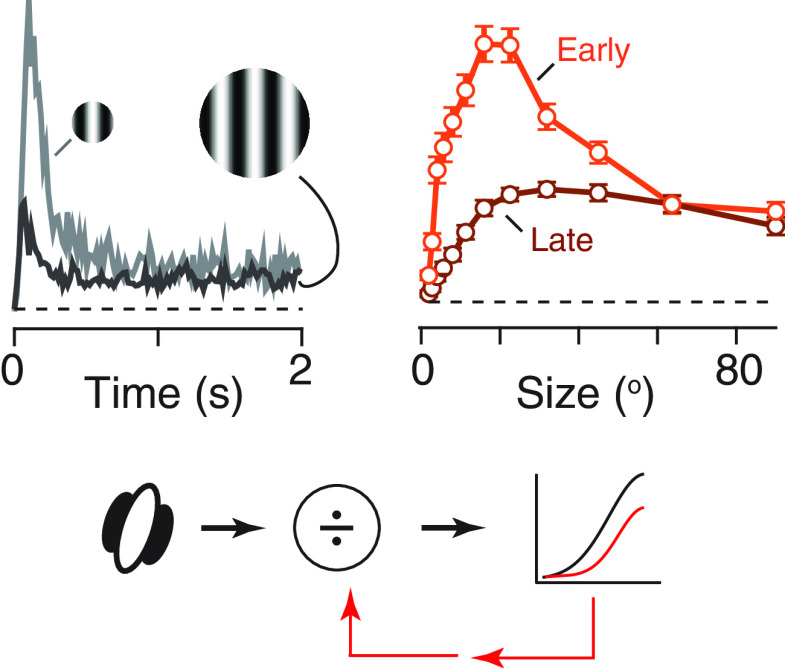

## Significance Statement

Our senses provide enormous amounts of information, and the central nervous system needs to filter this information to focus on potentially important objects. Here, we study two visual mechanisms that might highlight unexpected or surprising objects for further analysis: surround suppression and adaptation. We show that both mechanisms work to filter the neural signals provided by the superior colliculus (SC), a midbrain area important for directing behavior. We also show that the two mechanisms are unexpectedly intertwined, endowing rich dynamics on neural signals at the first central stage of sensory processing. Finally, our results suggest a subpopulation of neurons that is specialized for signaling the presence of potentially important objects.

## Introduction

Unexpected objects are likely to be important for behavior, and predictable objects less important. Many aspects of the functional organization of the visual system can be explained by supposing that neuronal activity is suppressed when the image falling on a receptive field is predictable. For example, inhibitory inputs to retinal neurons can be thought of as providing predictions about the intensity of the image over the receptive field, suppressing responses unless the intensity deviates from those predictions (Srinivasan et al., 1982). The functional consequences of this predictive inhibition are the classical center-surround organization and transient responses of receptive fields in the retina and its targets (Hartline, 1940; Kuffler, 1953; Barlow, 2001).

In the classical model of an early receptive field, inhibition provides predictions about the average intensity of the image over the receptive field, but not the variance, or pattern, of intensity in that image. Two additional mechanisms are needed to explain how responses to predictable patterns are suppressed. Spatial interactions (often called surround suppression) can suppress responses when the pattern over the classical receptive field (CRF) is similar to that in the surrounding region. Temporal interactions (often called adaptation) can suppress responses when the pattern is similar over time. The spatial and temporal suppression are thought to reflect the action of “gain controls,” mechanisms that regulate the responses generated by the CRF (Shapley and Victor, 1978; Bonds, 1989; Carandini and Heeger, 2011; Solomon and Kohn, 2014; Webster, 2015).

While most work on spatial and temporal gain controls has concentrated on visual cortex (Allman et al., 1985; Solomon and Kohn, 2014; rodent: Adesnik et al., 2012; Vaiceliunaite et al., 2013; Self et al., 2014; cat: Movshon and Lennie, 1979; Carandini and Ferster, 1997; monkey: Mayo and Sommer, 2008; Patterson et al., 2013), gain controls are also known to be important in the retina and early stages of central visual processing (rodent: Zhang et al., 2012; Jacoby and Schwartz, 2017; lagomorphs: Oyster and Takahashi, 1975; Smirnakis et al., 1997; cat: Sterling and Wickelgren, 1969; Jones et al., 2000; Bonin et al., 2005; Fisher et al., 2017; monkey: Solomon et al., 2002, 2004, 2006; Boehnke et al., 2011). In most animals the major target of the retina is the superficial layers of the midbrain superior colliculus (SC; homologous to the optic tectum; May, 2006; Ellis et al., 2016). The superficial layers of SC (SCs) project to, among other areas, the deeper layers of SC, which are important in organizing movements toward or away from potentially important objects (Dean et al., 1989; Basso and May, 2017; rodent: Comoli et al., 2012; Hoy et al., 2019). The receptive fields of superficial SC neurons are often remarkably selective for image features: for example, neurons in superficial SC of mouse can be tightly tuned for contour orientation, even in the absence of visual cortex (Wang et al., 2010; Shi et al., 2017). The receptive fields of neurons in superficial SC also show prominent surround suppression (rodent: Girman and Lund, 2007; Wang et al., 2010; Ahmadlou et al., 2017; Barchini et al., 2018; monkey: Davidson and Bender, 1991) and adaptation (Dutta and Gutfreund, 2014; monkey: Boehnke et al., 2011). How gain controls influence the response of SC neurons is less clear, particularly in awake animals. For example, we do not know whether surround suppression and adaptation’s effects are ubiquitous, whether they are independent, or how they interact.

Here, we made extracellular recordings from the superficial layers of SC in awake mice. We characterized surround suppression from the response to drifting gratings of varying size, and characterized adaptation from the time course of the response to drifting gratings of optimal size. We find profound impact of surround suppression and adaptation in many but not all neurons, and show that neurons with strong suppressive surrounds are also more susceptible to adaptation. Further, the suppressive surrounds themselves are susceptible to adaptation, and adaptation’s effects reveal at least two components of suppression, an untuned component that is adaptable, and a tuned component that is less adaptable. The selectivity of the tuned component was not static, but flexible: that is, suppression depended on what was shown to the receptive field, and was stronger when the stimulus over the surround matched that over the receptive field. The spatial and temporal gain controls may therefore allow neurons in the superficial SC the capacity to dynamically signal unexpected events in either space or time.

## Materials and Methods

### Ethical approval

All animal procedures were performed in accordance with the United Kingdom Animals Scientific Procedures Act (1986). Experiments were performed at University College London in accordance with its animal care committee’s regulations, under personal and project licenses released by the Home Office following appropriate ethics review, and in accordance with the ethical policy under which *eNeuro* operates.

### General

Adult C57BL/6 male mice (8–12 weeks at the start of experiments, 20–35 g) were obtained from Charles River Laboratories. Animals were housed with *ad libitum* food and water, on an inverted 12/12 h light/dark cycle. Measurements were obtained during the dark phase. To prevent damage to implanted devices, animals were singly housed after the preparatory surgeries described below.

#### Preparation for recordings

Anesthesia was induced with 3% isoflurane in O_2_ and the animal transferred to a stereotaxic apparatus. Anesthesia was subsequently maintained with 1–1.5% isoflurane in O_2_, and adjusted as necessary by monitoring the breathing rate and absence of reflex responses to paw pinch. The scalp was retracted and a craniotomy was made in one hemisphere, centered 3.5–3.7 mm posterior to bregma, 0.7–1.1 mm lateral to the midline suture. A metal head postfixed to the skull and a ground screw implanted over frontal cortex. In six animals the brain was covered with a layer of Kwik-Cast Sealant (WPI), which was replaced with artificial cerebrospinal fluid (Bio-Techne Ltd) during recording sessions; in these cases, recordings were subsequently made using quartz/platinum-tungsten electrodes (Thomas Recordings; impedance 4–5 MΩ) or tetrodes (impedance 0.5–0.8 MΩ). In two animals the dura mater was instead removed and a 16-channel microdrive (arranged as four tetrodes; Axona Ltd) was implanted. Animals recovered from surgery for at least one week and were then habituated to head-restraint before recordings started. Typical duration of a recording session was 90–120 min. At the end of the experiments, animals were euthanized by overdose of sodium pentobarbital intraperitoneal.

#### Recordings and spike sorting

The analog signal from each electrode was amplified and filtered (0.3 kHz −7/10 kHz), then digitized and recorded at 48 or 44 kHz. All recordings obtained at one site on 1 d were analyzed together. Putative single units were identified off-line using Plexon Offline Sorter (version 3.3.2, for single electrode recordings) or KlustaSuite (Rossant et al., 2016). Single units were identified by clustering in principal component (PCA) space, followed by manual inspection of spike shape, auto-correlograms and cross-correlograms. In no putative single unit did the fraction of interspike interval (ISIs) under 0.5 ms exceed 2%.

#### Visual stimuli

Visual stimuli were generated using Expo (P. Lennie) on an Apple Macintosh computer, and presented on a LCD monitor (Iiyama ProLite E1890SD, mean luminance 35–45 candela/m^2^; 38 cm wide, 29 cm high) refreshed at 60 Hz and displaying a gray screen of the mean luminance, positioned 20 cm from the animals’ eye. The monitor was γ-corrected by measuring the luminance of the red, green and blue elements with a photometer (Konica Minolta, Chroma meter CS-100A). Neural recordings were aligned to the visual stimulus by the output of a photodiode scanning a small corner of the stimulus monitor shielded from the animal. The coarse location of receptive fields was manually identified and the monitor location adjusted to approximately center them while making the monitor normal to the animal. Receptive field position estimates were subsequently refined by on-line analysis of responses to “sparse-noise,” where black or white squares (size 15°; spacing 7.5°, duration 0.2 s) were presented pseudo-randomly at each location of a 9 × 9 grid centered in the monitor, so that the squares spanned 75° × 75° of visual space. Our recordings were made from a variety of elevations in the nasal visual field, or from the lower temporal visual field, and were not distributed sufficiently for us to characterize the relationship between receptive field location and functional properties. We did not correct the display for the distortions in visual angle or changes in illumination that the short viewing distance produces at the edges of the monitor. Stimuli lasted for 2 s with an interstimulus interval of 0.5 s. Each set of stimuli included a blank condition (during which the screen was held at the mean luminance) from which “spontaneous” or maintained firing rates were estimated. Each set of stimuli was presented in pseudo-randomized order for 3–15 repetitions.

In some experiments we presented a drifting sinusoidal grating in a circular patch of varying diameter (2−90°), outside of which the screen was held at the mean luminance. The spatial and temporal frequency of the gratings was determined by initial measurements at each site. We used a spatial frequency near the optimal for the neurons under consideration (usually 0.05 cycles/°; μ 0.09, range 0.04–0.30); temporal frequency was usually 4 Hz (μ 3.5 Hz; 0.7 Hz, *n* = 2 units; 2 Hz, *n* = 33; 4 Hz, *n* = 56; 7.5 Hz, *n* = 6); Michelson contrast was 0.99 (hereafter normalized to 1.0) unless varied. In additional experiments, we presented a central patch of grating with a surrounding (abutting) annular grating. The central patch was of fixed size, and of the spatial and temporal frequency defined above; the annular grating was of the same spatial frequency, and a temporal frequency 0.5 Hz higher. In one experiment we varied the contrast of the annular grating, and in another experiment, we varied the orientation/direction of the annular grating. Each set of stimuli included trials in which the central patch or an annular grating was presented in isolation. Measurements were drawn from a large set of units, some of which have been reported previously (De Franceschi and Solomon, 2018).

### Data analysis

#### Analysis

Offline analysis was performed in the MATLAB environment (R2019a; The MathWorks). Peristimulus time histograms (PSTHs; bin width 0.016 s) were constructed for each trial, from which we extracted the mean firing rate. Unless stated, we define response as stimulus evoked activity, that is, the change in activity from that measured during presentation of a blank screen (the spontaneous or maintained firing rate).

#### Inclusion criteria

We considered neurons visually responsive if their maximal response exceeded the maintained rate by at least 1.5 SD of that rate, and further required that their response exceed two impulses/s in the relevant analysis. We also required the center of a units’ receptive field (estimated from responses to the sparse-noise stimulus) to be within 10° of the stimulus center.

#### Size tuning

To characterize the dependence of response on the size of a grating patch we assumed that both the CRF and a suppressive surround could be described by concentric circular Gaussians (Cavanaugh et al., 2002a). The excitatory CRF (*Le*) to a grating of diameter *d* is proportional to the integrated volume of a Gaussian:

(1) $$L_{e}\left(d\right)=\frac{2}{\sqrt{\pi }}\overset{d}{\underset{0}{\int }}e^{-{\left(x/r_{e}\right)}^{2}}dx,$$

where *r_e_* is the width of the Gaussian envelope. A similar expression can be derived for the larger surround Gaussian (*Li*). We assumed that the surround has divisive influence on the activity of the CRF (Sceniak et al., 2001; Cavanaugh et al., 2002a), such that response is:

(2) $$R\left(d\right)=\frac{K_{e}L_{e}(d)}{1+K_{i}L_{i}(d)},$$

where *K_e_* and *K_i_* are, respectively, the excitatory and the suppressive gains. We found the set of parameters that maximized the log-likelihood (*LL*) of the model given the responses (El-Shamayleh and Movshon, 2011) using the MATLAB function *fmincon*. We compared the model *LL* to an upper bound (*LLu*; obtained by fitting the responses to themselves) and a lower bound (*LLl*; obtained by fitting the responses to the average response across all stimuli). The normalized log-likelihood [*LLn* = (*LL* – *LLl*)/(*LLu* – *LLl*)] was used to decide whether to include the resulting model parameters in subsequent analyses (*LLn* ≥ 0.5). In addition to the parameters described above we included an additional parameter that allowed for a maintained discharge rate, and included in the set of responses to be modelled the activity during presentation of a blank gray screen. We estimated the preferred size from the model fit as the smallest size reaching 95% of the maximal response.

#### Suppression index (SI)

To quantify the suppression observed in size-tuning curves we calculated a SI as:

(3) $$SI=100\times \frac{R_{opt}-R_{large}}{R_{opt}},$$

where *R_opt_* is the response amplitude at the preferred size and *R_large_* is the response amplitude at the largest tested size; both were extracted from the best predictions of the model above. We used the same expression to quantify suppression in center-surround experiments, substituting *R_opt_* with the response to a central patch alone, and *R_large_* with the response to the relevant combination of central patch and annular grating.

#### Adaptation index (AI)

We calculated an AI to characterize the change in response to a stimulus over time:

(4) $$AI=100\times \frac{R_{early}-R_{late}}{R_{early}},$$

where *R_early_* and *R_late_* are the average evoked activity during the first and last 0.5 s of stimulus presentation, respectively (the stimuli lasted for 2 s).

#### Orientation/direction tuning

We calculated the direction tuning of stimulus-evoked responses or SI as the amplitude of the vector sum of responses or SI to different directions:

(5) $$gDSI=\frac{\sum^{}R_{\theta }e^{i\theta }}{\sum^{}R_{\theta }},$$

where *R_θ_* is the response to a grating of direction *θ*. A global index of orientation selectivity is defined in the same way, but after doubling *θ*. The preferred direction or orientation is the angle of the relevant vector sum. We used the same expression to orientation/direction tuning of surround suppression by substituting *R_θ_* with the SI (SI*_θ_*) measured for an annular grating of direction *θ*.

### Statistics

All statistical comparisons were performed in MATLAB. Correlations are the Pearson’s correlation coefficient, *r*. Statistical tests are Student’s paired *t* tests unless noted.

## Results

Most models of receptive fields early in the visual pathway suppose that the signals of different photoreceptors are given appropriate weight (which may be excitatory or inhibitory) and then summed to provide a receptive field that drives spiking output. These models can be used to characterize neurons with center-surround receptive fields as well as those neurons with more complex response properties, such as orientation tuning (Cheong et al., 2013). These models are, however, unable to explain why the response of neurons often depends on the structure of the image beyond the receptive field, or the previous history of stimulation. Explaining these dependencies requires supposing additional spatial and temporal gain controls, which regulate the sensitivity of the receptive field.

**Figure 1.**
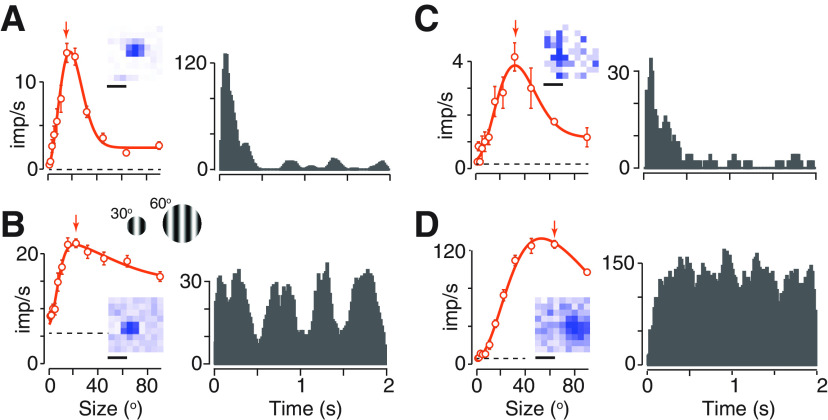
Expression of spatial and temporal gain controls in neurons in SC of awake mouse. ***A–D***, Responses of four representative neurons. The left panel in each case shows the average firing rate of the neuron during a 2-s presentation of a circular patch of drifting grating at a spatial frequency near the preferred for the neuron (0.05–0.07 cycles/^o^), and centered on the receptive field. The right panel in each case shows the PSTH (bin width 0.016 s) during presentation of a patch of grating near the preferred size for that neuron, which is indicated by the arrow in the left panel. Dashed horizontal lines show the maintained rate in absence of patterned visual stimulus. Solid line shows the predictions of the size-tuning model described in Materials and Methods (Eq. 2). Error bars show ±1 SEM across trials. The insets show a spatial map of responses (white indicates no activity and darker colors indicate stronger responses) to a black square, 15° wide, flashed at each of 81 positions on the monitor. Calibration bars are 26°. The schematic below panel ***A*** shows the relative size of two patches of grating and how a grating of 0.05 cycles/^o^ would appear in each of them.

The presence of spatial gain controls can be established by measuring the tuning of neurons to the size of a pattern. We therefore varied the diameter of a patch of drifting grating that was centered on the receptive field of the neuron under study (Fig. 1*A–D*). The response of most neurons was suppressed as the grating extended beyond the receptive field and into the surrounding region, showing the presence of a spatial gain control, or suppressive surround. The presence of temporal gain controls can be established from the time course of response to a visual stimulus. All neurons responded robustly at the onset of a small stimulus. In some neurons, the response was sustained throughout the stimulus duration (Fig. 1*B*,*D*), but in others, it was rapidly suppressed (Fig. 1*A*,*C*). This adaptation effect shows the presence of a temporal gain control.

### Prevalence of spatial and temporal gain controls

**Figure 2.**
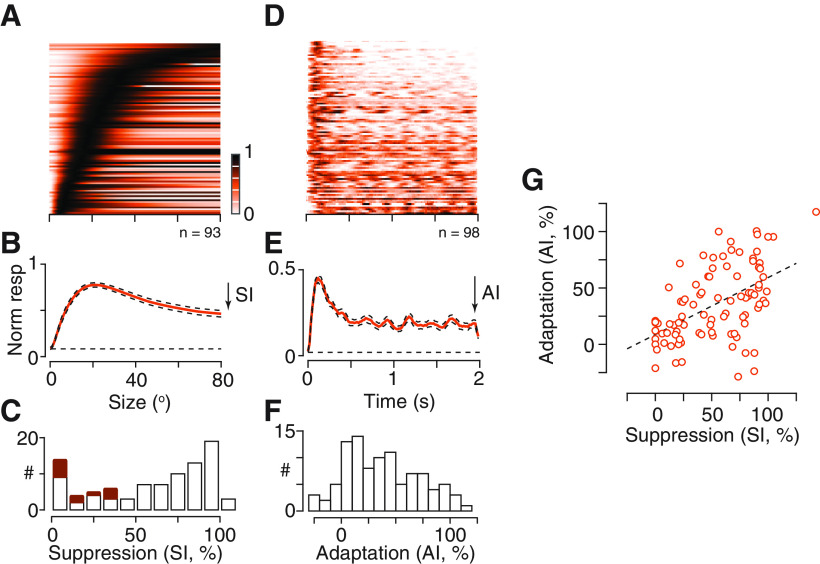
Correlated variability in surround suppression and adaptation effects. ***A***, Population size-tuning for patches of drifting gratings. Each row of the image shows the predictions of the size-tuning model for a single neuron (as in Fig. 1), normalized to its maximum response. Only units in which the normalized log-likelihood of the model was at least 0.5 are shown. The units are ordered, from bottom-to-top, by the preferred size. ***B***, Mean size-tuning for a drifting grating, obtained by averaging across the rows in ***A***. Dashed lines show ±1 SEM across neurons. Dashed horizontal line shows the maintained rate, normalized to the unit’s maximum visual response before averaging. Arrow indicates the definition of the SI, which is the proportional reduction in response from a grating of preferred size to a large grating. ***C***, Distribution of the SI across the population of units in ***A***. The filled bars show SI for neurons with preferred diameter >30°. ***D***, Population time course for drifting gratings of preferred size. The units are ordered, from bottom-to-top, by the AI. Color bar as in ***A***. ***E***, Mean time course for a drifting grating, obtained by averaging across the rows in ***D***. Conventions as in ***B***. Arrow indicates the definition of the AI, which is the proportional reduction in response from the first 0.5 s to the last 0.5 s. ***F***, Distribution of the AI across the population of units in ***D***. ***G***, Comparison of the SI and AI in individual neurons (*n* = 93). Dashed line is the best linear fit to the data.

We characterized the impact of the spatial gain control as the proportional reduction in response to a large grating (a SI; Eq. 3). Here, values of 0 indicate neurons in which there was no discernible suppression at large sizes, while values of 100 indicate neurons that only responded to small stimuli, and were completely suppressed by larger ones. On average this SI was 76.9% (median 70.4, SD 147.9, *n* = 93; Fig. 2*A–C*), but there was substantial variability across the population of neurons. The absence of suppression in some neurons might arise if their receptive fields are very large, and the gain control was beyond the extent of the stimulus monitor. Our sample included neurons that responded best to the largest grating we could produce, and Figure 2*C*, filled bars, shows that neurons preferring large gratings (diameter >30°) showed little suppression. Most neurons, however, preferred gratings 10–30° in diameter (geometric mean 16.3°, median 15.6°, *n* = 93), and in many of these neurons, we saw little suppression although the preferred size was well within the monitor gamut. The measurements above were obtained for patterns of high contrast. To establish the sensitivity of suppression in a sample of neurons we measured the response to a patch of grating of optimal size, and varied the contrast of an annular grating (data not shown). In these neurons, suppression at 25% annulus contrast was on average 21.6% (SD 15.7, *n* = 12), about half that at 100% annulus contrast (38.3, SD 22.0; *p *=* *0.0139, paired Student’s *t* test). Spatial gain controls can therefore be engaged at low image contrast, and their impact increases with contrast.

To characterize temporal gain control independently from spatial gain control we examined responses to a patch of drifting grating of the preferred size for the neuron under study. We measured the impact of temporal gain controls as the proportional reduction in response from early (the first 0.5 s) to late (the last 0.5 s) time points, producing an AI (Eq. 4) similar to the SI above. On average, later responses were suppressed by 37.4% (median 36.0, SD 33.3, *n* = 98; Fig. 2*D–F*), but as for surround suppression, we saw substantial variability across the population of neurons (Fig. 2*F*). This variability in AI was not explained by variation in temporal frequency of the grating (2 Hz: μ 33.1%, SD 23.8%, *n* = 33; 4 Hz: μ 38.4%, SD 36.1, *n* = 56). In a sample of neurons we measured AI for a small patch of grating at low or high contrast (data not shown): the AI at 25% contrast was on average 47.9% (SD 35.0, *n* = 27), if anything stronger than at 100% contrast (25.0%, SD 72.1, *n* = 28; *p *=* *0.13, paired Student’s *t* test). Thus, temporal gain controls are also sensitive to low image contrast.

The substantial variability in surround suppression and adaptation’s effects raises the question of whether the spatial and temporal gain controls are co-expressed in individual neurons. To establish this, we compared the shape of the size-tuning curves for drifting gratings (provided by the SI) and the time course of response for small patches of drifting grating (provided by the AI). We found strong surround suppression in neurons that showed strong adaptation effects (Fig. 1*A*,*C*) and weak surround suppression in neurons that showed weak adaptation effects (Fig. 1*B*,*D*). Consequently, when we compared the index of surround suppression (SI) and the index of adaptation (AI), we found a positive correlation (*r *=* *0.51, *p *<* *0.00001, Pearson’s correlation coefficient; Fig. 2*G*). Spatial and temporal gain controls therefore appear to be co-expressed in individual neurons.

Many, but not all, neurons in SC are tuned for the orientation or motion direction of a grating. We therefore asked whether this tuning might predict the expression of surround suppression or adaptation effects (data not shown). We found little relationship between adaptation’s effects (AI) and global measures of orientation or direction tuning (respectively, *r* = 0.08, *r* = 0.16; *p* = 0.47, *p* = 0.17; *n* = 78). We found more of a relationship for surround suppression (SI; respectively, *r* = 0.33, *r* = 0.36; *p* = 0.0028, *p* = 0.0014). Units with little surround suppression were usually weakly tuned for orientation or direction, while units with strong surround suppression included units with a range of tuning for orientation/direction.

### Tuned and untuned contributions to spatial gain controls revealed by adaptation

**Figure 3.**
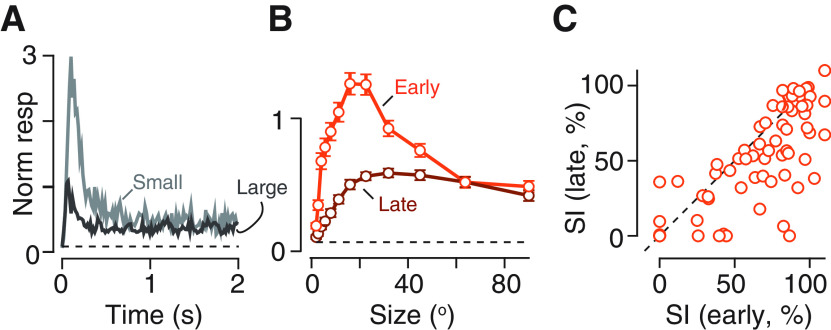
Surround suppression is susceptible to adaptation. ***A***, Time course of population response for gratings for small (20° diameter) and large (90°) patches of drifting grating. Responses were normalized to the mean response across all patch sizes (most of which are not shown) before averaging (*n* = 98). Dashed horizontal line shows the maintained rate in absence of patterned visual stimulus, normalized in the same way. Error bars are omitted for clarity. ***B***, Size-tuning for early (0–0.5 s) and late (1.5–2 s) response, normalized in the same way as ***A***. Error bars are ±1 SEM across neurons that passed criteria for inclusion (early: *n* = 92; late: *n* = 80). ***C***, Comparison of SI for early and late response (*n* = 73). Dashed line shows the unity line. Points falling below the line indicate neurons in which suppression was stronger in the early response than in the late response.

Inspection of PSTHs for small and large stimuli showed that responses to small stimuli were more transient, that is, adaptation’s effects were stronger for small stimuli (Fig. 3*A*). This suggests that spatial and temporal gain controls interact in shaping neural response. We characterized this interaction by generating size tuning curves for early and late responses. We found less surround suppression at late time points (Fig. 3*B*), and our index of suppression consequently reduced over time (on average from 69.0% to 51.2%, *n* = 73, *p *<* *0.00001, paired Student’s *t* test; Fig. 3*C*). The reduced suppression at late time points suggests that surround suppression is also adaptable.

**Figure 4.**
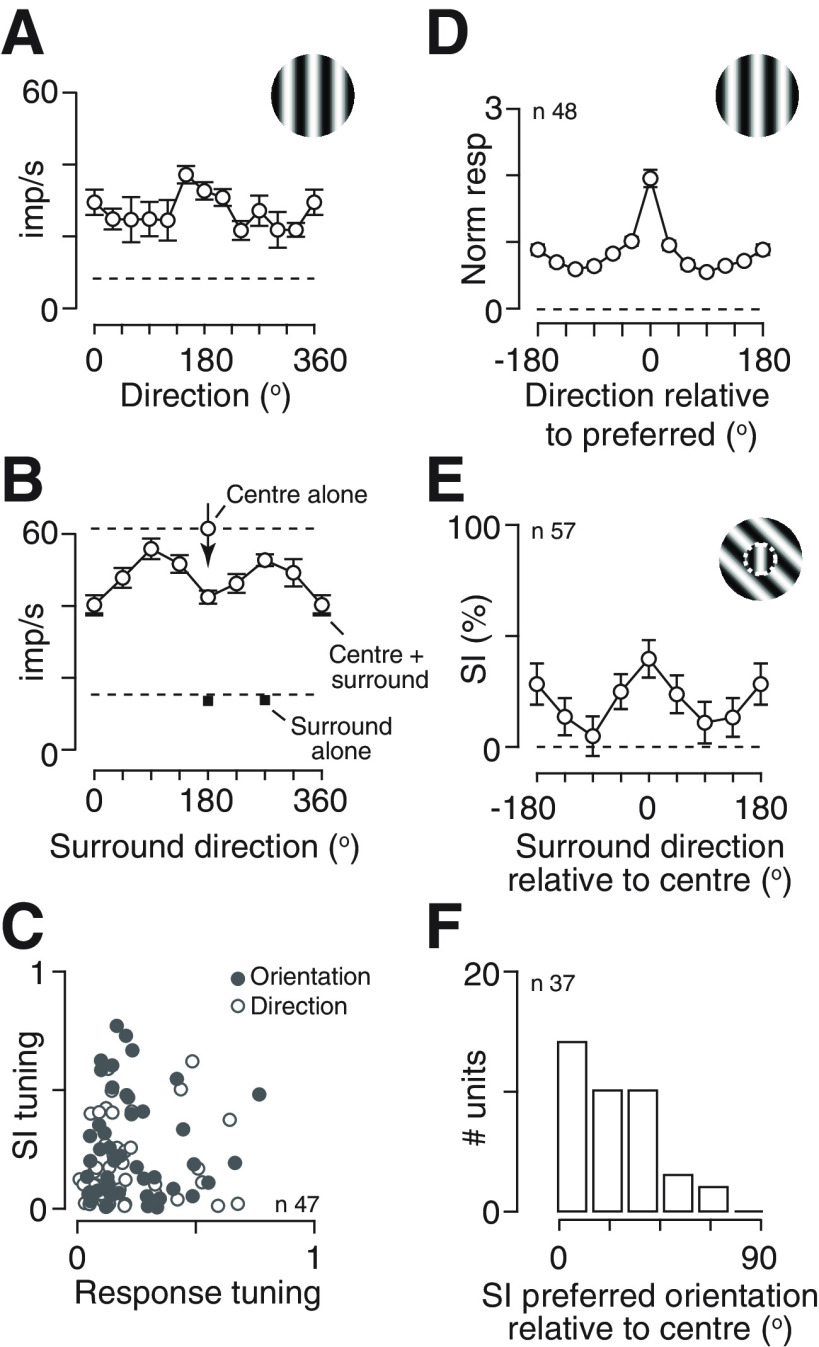
Tuning of surround suppression in SC of awake mouse. ***A***, ***B***, Response of an example neuron. ***A***, Tuning of spiking activity evoked by a large patch of drifting grating (45° diameter) of varying orientation/direction. Dashed horizontal line shows the maintained rate in absence of patterned visual stimulus. ***B***, Tuning of suppression induced by an annular grating of varying orientation/direction. Responses are shown for presentation of a 15° patch of drifting grating (upper dashed line, “center alone”) of direction 180°, the same stimulus when abutted by an annular grating of outer diameter 80° (“center+surround”), and two of the annular gratings presented in absence of the center grating (“surround alone”). The lower dashed horizontal line shows the maintained rate in absence of patterned visual stimulus. A SI can be calculated for each annulus direction as the proportional reduction in response from the “center alone” stimulus to the relevant “center+surround” stimulus. Error bars in ***A***, ***B*** are ±1 SEM over trials. ***C***, Comparison of tuning for spiking activity (abscissa) and suppression (ordinate). Each unit contributes two points: the open symbols indicate a global measure of direction tuning (Eq. 5) and the filled symbols indicate a similar measure of orientation tuning. ***D***, ***E***, Population averages. ***D***, Population average spiking activity evoked by a large grating, after aligning each neuron to its preferred direction, subtracting the maintained rate, and then normalizing by the mean response across all stimuli. ***E***, Average SI, obtained as in ***B***, after aligning each neuron to the direction of the central grating patch. Dashed horizontal line shows an SI of zero. Error bars in ***D***, ***E*** are ±1SEM over neurons. ***F***, Distribution of preferred orientation of suppression, relative to the orientation of the center grating, in units in which the preferred orientation could be defined (orientation tuning index >0.1). A relative orientation of zero indicates neurons in which the most suppressive stimulus was the same orientation as the center; a relative orientation of 90 indicates neurons in which the most suppressive stimulus was orthogonal to the central stimulus. Schematics in panels ***A***, ***D***, ***E*** are not to scale.

If adaptation changes the sensitivity of surround suppression, it may also change the tuning of surround suppression. Previous work shows that surround suppression in mouse SC can be sensitive to the orientation and/or direction of a pattern (Ahmadlou et al., 2017; Barchini et al., 2018). We confirmed that suppression in SC was usually strongest when the orientation and direction of the annular grating matched that over the receptive field (Fig. 4*A*,*B*). In many neurons (for example, the unit in Fig. 4*B*), and in the population average (Fig. 4*E*), suppression was similar for either direction of motion of a parallel annular grating. In other neurons suppression was clearly stronger when the direction of the annular grating also matched that in the central patch, and in others surround suppression was untuned. We therefore asked whether suppression was more tuned in neurons in which spiking response (Fig. 4*A*,*D*) was also strongly tuned. We used a global index of orientation or direction selectivity (see Materials and Methods) to compare the tuning of neuronal responses to a single large grating, with the tuning of suppression elicited by the annular gratings. In both cases, values of 0 indicate no tuning, while values of 1 indicate spiking response or suppression for only one stimulus. There was little correlation (*r* = −0.02/0.09, *p *=* *0.90/0.53, Pearson’s correlation coefficient; Fig. 4*C*): suppression was often tuned even when spiking response was untuned, and vice versa. Nevertheless, in neurons where the tuning of suppression was strong enough to define a preferred stimulus (tuning index >0.1), the preferred orientation/direction of suppression was generally aligned with the stimulus shown in the central patch (Fig. 4*F*).

**Figure 5.**
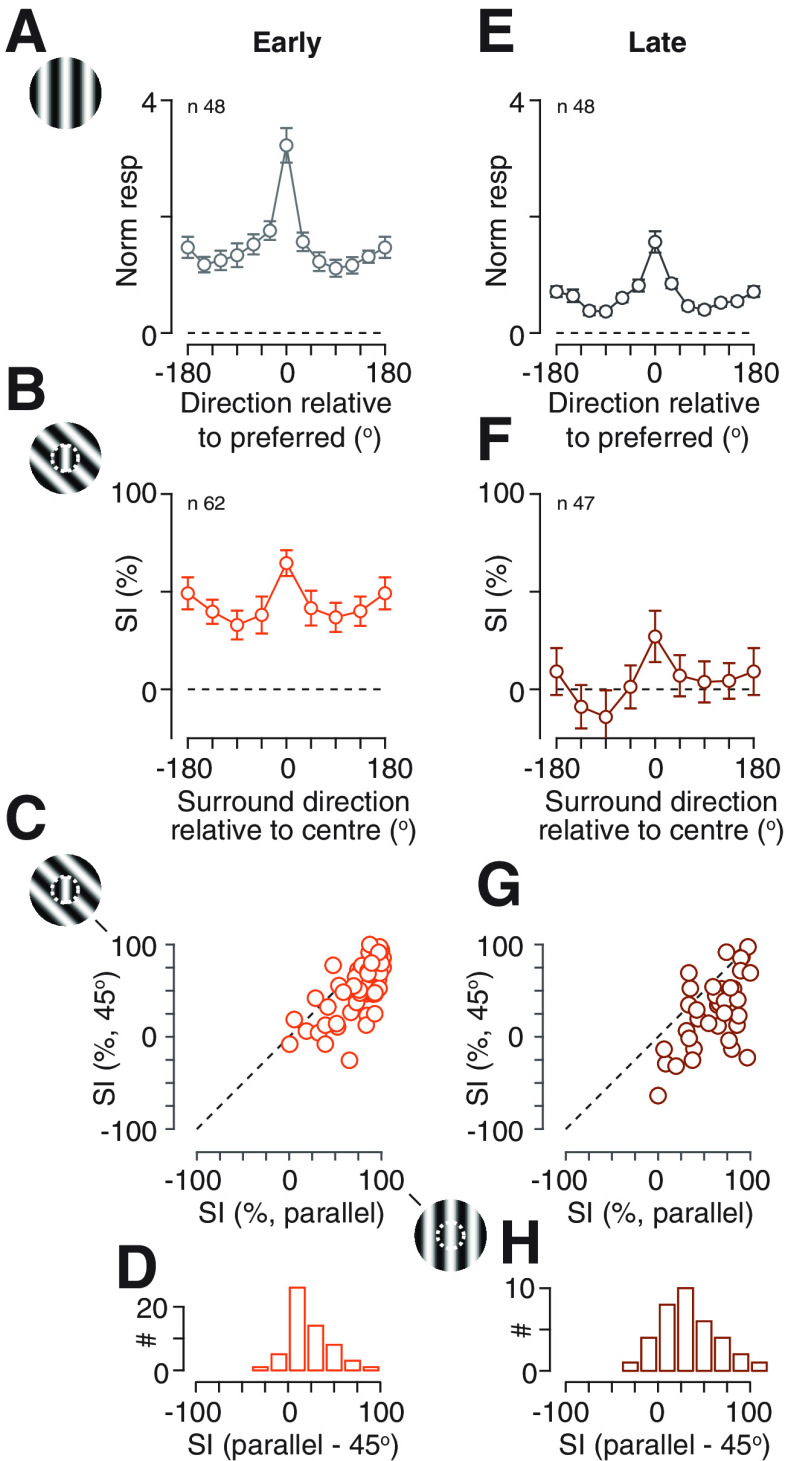
Impact of adaptation on tuning of spiking activity and surround suppression. ***A–D***, Responses in early (first 0.5 s) time points. ***A***, Population average tuning of spiking activity evoked by a large grating. Conventions as in Figure 4*D*. ***B***, Population average tuning of suppression induced by annular grating. Conventions as in Figure 4*E*. ***C***, Comparison of SI for annular gratings that match the direction of the central grating (“parallel,” abscissa), or are tilted by 45° (ordinate). SI for the latter was averaged across both possible directions of tilt. Dashed lines are the unity line. ***D***, Distribution of the difference in suppression for the two annular gratings. Positive indices indicate more suppression by a parallel annulus than a tilted annulus. ***E–H***, Same ***A–D***, but for late (last 0.5 s) time points. Schematics in panels ***A–C*** are not to scale.

To establish whether the tuning of suppression is changed by adaptation, we measured the tuning of suppression in the first 0.5 s following the onset of the stimulus, and in the last 0.5 s. The population average showed strong suppression at early time points, but this suppression was only weakly tuned for annulus orientation (Fig. 5*B*). At later time points the overall strength of suppression was reduced and was largely confined to gratings of the same orientation/direction as the central patch, resulting in increased selectivity of suppression (Fig. 5*F*). To illustrate how tuning changed in individual neurons we compared suppression for annular gratings of the same orientation and motion direction as the central patch, with that for gratings tilted by 45° (average of ±45°; Fig. 5*C*,*G*). Parallel gratings generated stronger suppression at both time points, but their advantage was less pronounced at early (parallel gratings generated μ 21.1% more suppression than tilted gratings, SD 22.3, *n* = 58; Fig. 5*C*,*D*) than late timepoints (μ 32.6%, SD 31.8, *n* = 36; Fig. 5*G*,*H*; *p = *0.0059, paired Student’s *t* test, for 35 units that could be characterized at both time points). We conclude that the overall strength of surround suppression reduces over time, and the selectivity of suppression increases.

The pattern of results in Figure 5*B*,*F* might be explained if spatial gain controls constitute two mechanisms, one that is narrowly tuned for orientation/direction and less susceptible to adaptation, and one that is more broadly tuned and more susceptible to adaptation. One potential source of suppression is the response of other neurons in SC and we therefore conducted similar analyses of spiking response to large drifting gratings (Fig. 5*A*,*E*). As for suppression, the population spiking response has both tuned and untuned components, and as for suppression the population spiking response reduced substantially at later time points, showing the presence of adaptation effects. This reduction in response was similar for the preferred grating and a grating tilted by 30° (*p = *0.0985, *n* = 34). Similar results were obtained if we compared responses to preferred and orthogonal gratings, examined direction tuning curves in a larger dataset including additional units (*n* = 155; data not shown), or compared the global orientation and direction selectivity indices. We conclude that adaptation’s effects on spiking activity in SC may be sufficient to explain why the overall strength of suppression is reduced at late time points, but additional mechanisms may be required to explain why the tuning of suppression increases at late time points.

### Flexible tuning of spatial gain controls

**Figure 6.**
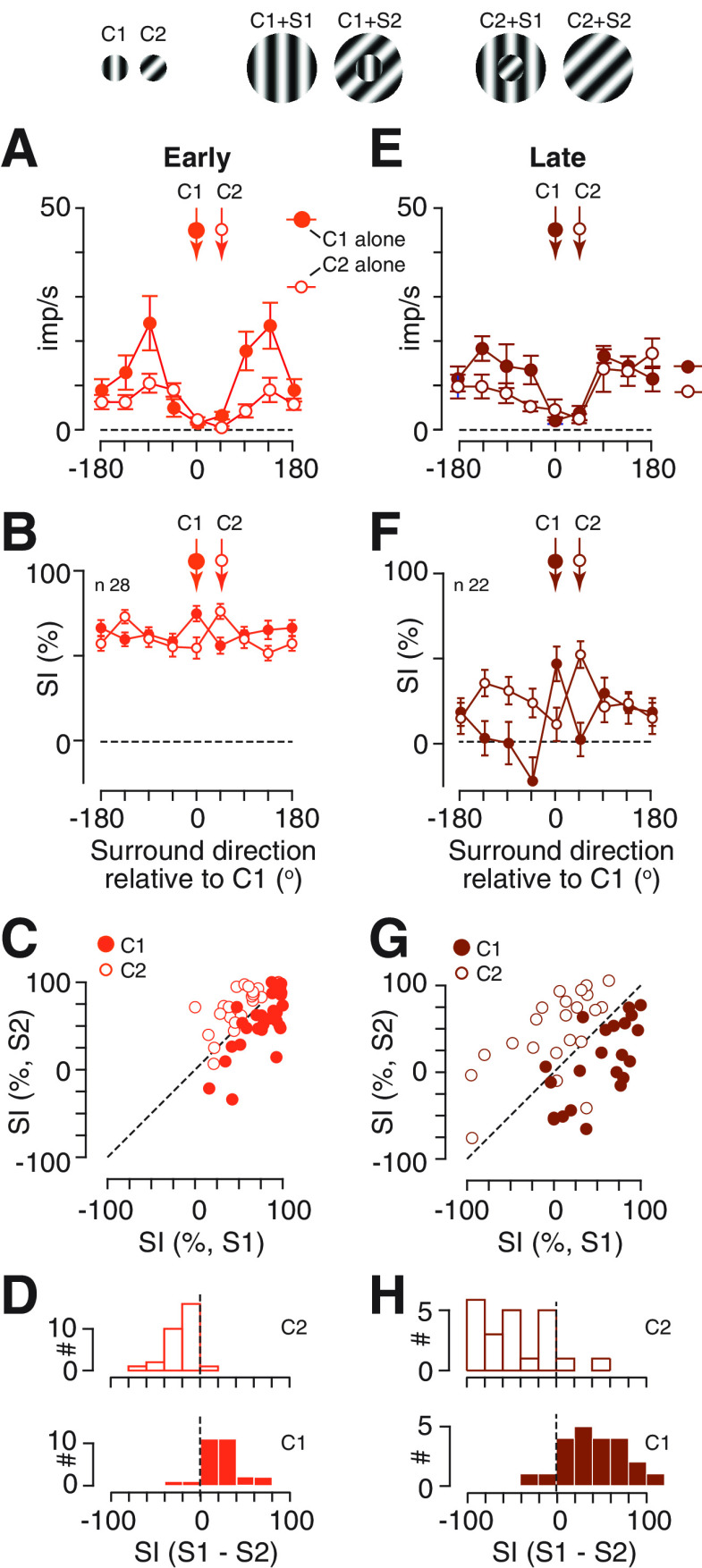
Adaptation’s effects magnify flexible surround suppression. ***A–D***, Responses in early (first 0.5 s) time points. ***A***, Spiking activity of an example neuron during presentation of a central stimulus either presented alone (C1, C2), or abutting an annulus of varying direction. Two measurements were made, first with a central grating near the preferred direction of spiking activity (C1) and then with a central grating tilted by 45° (C2). Error bars are ±1 SEM over trials. ***B***, Population average tuning of suppression for each of two central gratings (C1, C2). Conventions as in Figure 4*E*. ***C***, Comparison of suppression induced by pairs of stimuli. The abscissa shows the suppression induced by an annular grating (S1) that matched the direction of the central grating C1. The ordinate shows the suppression induced by an annular grating (S2) that matched the central grating C2. Points below the unity line (dashed line) indicate stronger suppression for S1 than S2. Filled circles show measurements obtained with C1: these generally lie below the unity line, indicating that when C1 is used, S1 is more effective than S2. Open circles show measurements obtained for C2: measurements are generally above the unity line, indicating that S2 is more effective than S1. This is the pattern of results expected if suppression is stronger when the stimuli over the center and surround are matched. ***D***, Distribution of the difference in suppression for each of the center gratings, C1 (lower) and C2 (upper). Positive indices indicate more suppression by S1 than S2. ***E–H***, Same as ***A–D*** but for late (last 0.5 s) time points. Example neuron in ***E*** is the same as that in ***A***. Schematics above ***A***, ***E*** are not to scale.

The tuning of spatial gain controls could either be static, or depend on the parameters of the stimulus over the receptive field. In other words, the tuning may be “fixed” or “flexible.” In a sample of neurons that were suppressed by annular gratings we therefore repeated the measurements after rotating the orientation/direction of the central patch by 45°. Suppression is relatively broadly tuned, and we therefore expected to see similar tuning curves for suppression across the two measurements. This was the case (Fig. 6*A*,*E*). Nevertheless, the most suppressive surround did depend on the orientation/direction of the central patch, at both early (Fig. 6*A*,*B*) and late (Fig. 6*E*,*F*) time points.

To establish how surround suppression depended on what was shown over the receptive field we focused our analyses on suppression evoked by the pair of annular gratings that matched the orientations/directions of the pair of gratings shown to the receptive field. The orientations of the central gratings over the receptive field are labeled C1 and C2 in Figure 6, and the annulus orientations that matched them are, respectively, S1 and S2. The analyses in Figure 6*C*,*G* show that annular gratings were relatively more effective when they matched the central patch. That is, when C1 was the central grating, suppression at S1 was stronger than suppression at S2 (Fig. 6*C*,*G*, points lie below the diagonal), and when C2 was the central grating the pattern was reversed (points lie above the diagonal). To compare the suppression that was evoked by S1 and S2 at each of the center orientations we calculated the difference in suppression for the two conditions, that is, for C1 we calculated SI_S1_ – SI_S2_, and for C2 we also calculated SI_S1_ – SI_S2_. This subtraction collapsed the data along the diagonal in Figure 6*C*,*G* while preserving sign, and produced the distributions in Figure 6*D*,*H*.

The advantage of matched annular gratings appeared to increase with time: suppression indices lie further away from the diagonal at later time points (Fig. 6*G*) than they do at early time points (Fig. 6*C*). Similarly, the distributions in Figure 6*H* lie further from 0 than do the distributions in Figure 6*D*. To provide a statistical comparison across early and late time points, we computed an additional index, [MI = (SI_C1,S1_ – SI_C1,S2_) – (SI_C2,S1_ – SI_C2,S2_)] for each unit at each time point. If suppression was fixed, and thus the same for any particular annulus orientation, regardless of center orientation, then this MI should be 0, but it was not (early: μ 40.4, SD 33.1, *n* = 28; late: μ 85.4, SD 60.1, *n* = 22). Comparison of the indices at early and late time points, for units that responded in both, showed that the index increased at later time points (*p *=* *0.000459, *n* = 22; paired Student’s *t* test). We conclude that the tuning of suppression in SC can be flexible, and that this flexibility is most apparent at later time points, when the untuned gain control is less effective.

## Discussion

### Functional impact of temporal gain controls

Adaptation’s effects allow neurons to adjust their activity to the recent stimulus history. The reduction in response to a stimulus that is unchanging might be used by neurons to better signal changes over time. We have shown that many neurons in the mouse SC show adaptation effects: responses are characterized by a large initial response that is quickly suppressed. This suppression cannot be explained by retinal light adaptation, because we presented drifting gratings, where the spatial pattern is constantly modulated.

The timescales of adaptation effects that we have characterized are on the order of 10–100s of milliseconds, shorter than most earlier characterizations of adaptation effects in SC, which were primarily conducted in anaesthetized animals (e.g., rabbit: Horn and Hill, 1966; monkey: Cynader and Berman, 1972; mouse: Dräger and Hubel, 1975; pigeon: Woods and Frost, 1977). That work emphasized a large and long-lasting suppression of response (often called habituation). The habituation was strongest in the intermediate and deeper layers of the SC, but it has also been reported in the superficial layers (rabbit: Oyster and Takahashi, 1975; cat: Binns and Salt, 1995). n anaesthetized rat, more rapid adaptation effects were seen in superficial neurons for flashes of bright spots on a dark background (Bytautiene and Baranauskas, 2018), but those effects may have included a contribution of light adaptation. Our stimuli were interleaved, and were preceded by other sets of stimuli, so it is difficult to establish the effect of long-term habituation from these measurements. Nevertheless, we did not see a clear impact of position in the stimulus sequence on response amplitude, even when we only considered units with high adaptation indices (data not shown). Recordings in superficial layers of awake monkey also show lack of long-term adaptation effects (Goldberg and Wurtz, 1972), and more substantial short-term adaptation effects (Mayo and Sommer, 2008; Boehnke et al., 2011).

Previous measurements of adaptation’s effect in SC have often involved repeated presentation of a brief stimulus (Boehnke et al., 2011), whereas we measured response to a single, longer, continuous stimulus. While the two types of stimuli are likely to engage the same mechanisms, that does not mean they will have the same effect (Solomon and Kohn, 2014). The transients associated with repeated flashes may be more effective at driving the adaptive mechanism(s) and repeated presentations may therefore induce greater changes in activity. Alternatively, the periods of rest between the presentations may allow adaptive mechanisms to recover, and repeated presentations may therefore have less effect. Onset transients appear to be increasingly important for information processing as one ascends through the visual hierarchy (Tovée et al., 1993; Müller et al., 2001), so differences in adaptive responses to repeated and continuous presentations may be more pronounced in later visual processing. SC integrates early and later visual inputs, so comparison of adaptive responses to flashed and continuous presentation may be of interest.

Adaptation effects are prominent in retinal ganglion cell response, and likely first emerge in the bipolar cell input to ganglion cells (salamander: Chander and Chichilnisky, 2001; salamander/rabbit: Baccus and Meister, 2002; monkey: Solomon et al., 2004; guinea pig: Zaghloul et al., 2005; mouse: Marco et al., 2013). It is therefore probable that some of the adaptation effects that we see in SCs are inherited from the retinal input, but we are not aware of reports of retinal neurons that show the complete suppression of response that we often encountered in SCs. Additional mechanisms in SCs, potentially mediated by GABA_B_ receptors and metabotropic glutamate receptors, have been implicated in presynaptic and postsynaptic adaptation effects in SCs (cat: Binns and Salt, 1995; rat: Cirone and Salt, 2001), and these are likely to enhance or supersede adaptation effects inherited from retinal input. In addition, the sustained response was slightly reduced at large stimulus sizes (compare with Fig. 3*A*), while the initial transient was strongly reduced. Size-dependence of the sustained response has also been observed in SC of monkey (Chen and Hafed, 2018), although direct comparison is difficult because that work explored shorter time-windows and stimuli confined to the receptive field.

### Functional impact of spatial gain controls

Suppressive surrounds have been described in the SC of many species (cat: Sterling and Wickelgren, 1969; monkey: Cynader and Berman, 1972; Wurtz et al., 1980; rat: Girman and Lund, 2007; zebrafish: Del Bene et al., 2010; barn owl: Mysore et al., 2010; Zahar et al., 2012, 2018), including mouse (Wang et al., 2010; Gale and Murphy, 2014; Ahmadlou et al., 2017; Barchini et al., 2018). We show that in awake mouse surround suppression consists of at least two components, one that is weakly tuned and adaptable, and another that is more tuned and less susceptible to adaptation’s effects. The tuned gain controls appear to have flexible selectivity, such that the most suppressive surrounding stimulus is that which matches the stimulus over the receptive field.

Most types of mouse retinal ganglion cell send axons to the SCs (Ellis et al., 2016), and several of these are known to show surround suppression. One is the ON-OFF W3 cell (Zhang et al., 2012), thought to be a homolog of the “net convexity detector” cells in the frog retina (Lettvin et al., 1959) and the local edge detector (LED) cells first described in rabbit (Levick, 1967). But size sensitive responses are also found in “high-definition” (HD; Jacoby and Schwartz, 2017) as well as the direction-selective J and BD retinal ganglion cell classes, which also project to SCs (Kim et al., 2010). Thus, some of the surround suppression that we observe in SCs may be inherited from the retinal input. Yet while adaptation effects can reduce the amount of inhibition onto retinal ganglion cells (salamander/rabbit: Baccus and Meister, 2002; mouse: Wark et al., 2009; Marco et al., 2013; salamander: Kastner et al., 2019), we are not aware of reports of adaptation effects on suppression in retina at the time scale of the rapid adaptation that we see in SCs. This suggests that lateral interactions within SCs are a strong contributor to the surround suppression that we see, and the simplest conclusion is that adaptation reduces surround suppression in SCs because adaptation reduces spiking activity in SCs.

Networks in the superficial layers of mouse SC include inhibitory lateral interactions that suppress the activity of simultaneously activated neurons (Phongphanphanee et al., 2014). Local inhibition from “horizontal cells,” which respond to large stimuli (Gale and Murphy, 2014), may be particularly important in providing surround suppression (Gale and Murphy, 2016), while “narrow field” and “wide field” cells appear particularly susceptible to suppression (Gale and Murphy, 2014). Similar mechanisms for constructing size tuning have been described in the zebrafish optic tectum (Del Bene et al., 2010). In addition, the SC receives substantial input from visual cortex (May, 2006), although the role(s) of cortico-collicular input remain unclear, these projections modulate gain of SC neurons but their absence seems to have little effect on tuning properties (Wang et al., 2010; Zhao et al., 2014) or surround suppression (Ahmadlou et al., 2017), at least in mouse. Indeed, surround suppression in the SC may precede that in primary visual cortex (V1; monkey: White et al., 2017) and inactivation of SC can interfere with surround suppression in V1 of mouse (Ahmadlou et al., 2018).

Units that were not selective for pattern orientation/direction were also less likely to show strong surround suppression. This result may reflect a straightforward correlation in the two functional properties, or surround suppression may be important for constructing selectivity for orientation or direction. Regardless, controlling for the size of stimuli is likely to be important in characterizing, and therefore understanding, the mechanisms of orientation and direction tuning in SC.

We found that tuned surround suppression was less susceptible to adaptation than untuned suppression, with the consequence that suppression was more sharply tuned and more flexible in later activity. Our finding that at least some of the suppression in SCs is flexible is in accord with recent calcium imaging from SCs of anaesthetized mouse (Barchini et al., 2018). That work showed suppression by surrounding gratings of the same motion direction as a central patch, and facilitation by surrounds of the opposite direction, particularly in excitatory cells. The dynamics of calcium signaling make comparison of response time course difficult, but the initial spiking response, where we find weakly tuned suppression, may have contributed less to the calcium signal than the subsequent response, where we find more tuning of suppression and some facilitation. Our finding that tilted surrounds could even become facilitatory in the late phase of responses raises the possibility that the tuning of late suppression may in fact reflect tuned facilitation. Flexible suppression selectivity may therefore reflect input from neurons with large receptive fields that are sensitive to image continuity (if they provide tuned suppression) or sensitive to image discontinuity (if they provide tuned facilitation). These flexible mechanisms may arise in SCs or in its inputs. If they arise in SCs, then one candidate may be the horizontal cells. Regardless, mouse SCs is likely to be a useful model for understanding the mechanisms that enable flexible suppression of neural responses by spatial context (Coen-Cagli et al., 2015).

The functional properties of surround suppression in SCs are remarkably similar to that described for V1 in many mammals. First, surround suppression in V1 is often orientation-selective and direction-selective (mouse: Self et al., 2014; cat: Nelson and Frost, 1978; DeAngelis et al., 1994; Ozeki et al., 2004; monkey: Sillito et al., 1995; Levitt and Lund, 1997; Cavanaugh et al., 2002b; Webb et al., 2005; Henry et al., 2013), and that tuning selectivity can be flexible (Sillito et al., 1995; Cavanaugh et al., 2002b). Second, in V1 of mouse, monkey, and human, this tuned suppression is complemented by an untuned suppression (mouse: Self et al., 2014; monkey: Webb et al., 2005; Henry et al., 2013; human: Schallmo et al., 2019), some of which may be inherited from earlier processing (cat/monkey: Sillito et al., 1993; cat: Ozeki et al., 2004; Bonin et al., 2005; Naito et al., 2007; monkey: Solomon et al., 2002; Camp et al., 2009). Third, some components of surround suppression in V1 of monkey and human are susceptible to adaptation (Cavanaugh et al., 2002a; Wissig and Kohn, 2012; Patterson et al., 2013; Schallmo and Murray, 2016), although in monkey V1, the tuned components of suppression may be more sensitive to adaptation than the untuned components (Webb et al., 2005).

### Summary

**Figure 7.**
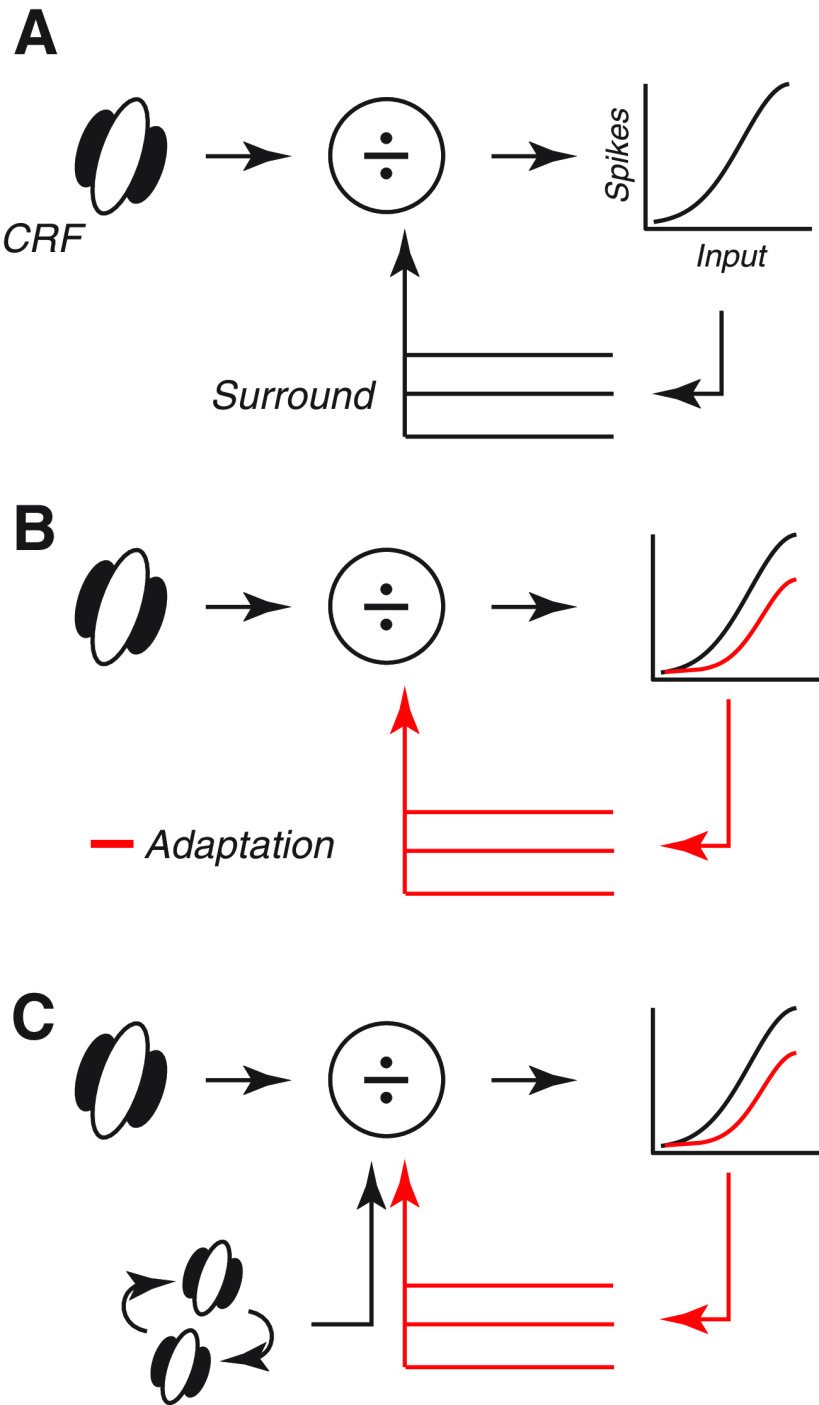
Descriptive model for interaction of spatial and temporal gain controls in SCs. ***A***, Standard model of receptive fields early in the visual pathway. The CRF filters the visual image, and its output is subject to a spatial gain control (surround) before driving spiking output. The surround is comprised of units with receptive fields similar to that of the CRF. ***B***, Adaptation’s effects reduce the response of the neuron under study, and the responses of neurons that contribute to the spatial gain control. ***C***, Addition of a second, less adaptable, component to the spatial gain control allows for preservation of suppression when the features of the image over the CRF matches that over the surround.

We have shown the presence of spatial and temporal gain controls in SCs of awake mouse and how they are distributed across neurons. Our results are consistent with the idea that these gain controls provide a predictive signal against which activation of the CRF is compared, thereby suppressing the response to predictable stimuli and highlighting unexpected ones. Our results can be accommodated by a layering of gain controls as illustrated in Figure 7. Figure 7*A* shows the standard model of early visual processing (Carandini and Heeger, 2011; Solomon and Kohn, 2014). The output of the CRF, which filters the visual image, is subject to a spatial gain control, or suppressive surround, before driving spiking activity. The suppressive surround is constructed from nearby neurons with similar characteristics. Adaptation’s effects can be thought of as changing the output function of the neuron, as shown by the red-line in Figure 7*B*. This accounts for the results in Figure 5, because similar neurons contribute to the surround, and the surround is therefore relatively broadly tuned for orientation/direction and susceptible to adaptation’s effects. To account for the advantage of surrounds that match the center stimulus (Fig. 6), and the apparent resilience of this suppression to adaptation’s effects, a second mechanism seems to be required (Fig. 7*C*). This is sensitive to the relationship between features over the CRF and surround and is less adaptable.

We also found that the strength of adaptation’s effects and strength of surround suppression were correlated among neurons. Our results therefore show that neurons characterized by a transient, adapting response are more likely to also be affected by spatial context, and may therefore signal the presence of unexpected objects in either the spatial and the temporal domain. This suggests the presence of a subpopulation of “novelty” or “saliency” neurons within the SC that are sensitive to unexpected events in the visual diet. Whether this functional subgroup has an anatomic correlate would be of interest. In monkey, the amplitude of the initial transient response in anatomically deeper visual-motor SC neurons, which receive direct input from the superficial purely visual neurons studied here, is known to be particularly important in the nature and latency of orienting behaviors such as saccades (Boehnke and Munoz, 2008; Chen and Hafed, 2017).

## Synthesis

Reviewing Editor: Tatiana Pasternak, University of Rochester Decisions are customarily a result of the Reviewing Editor and the peer reviewers coming together and discussing their recommendations until a consensus is reached. When revisions are invited, a fact-based synthesis statement explaining their decision and outlining what is needed to prepare a revision will be listed below. The following reviewer(s) agreed to reveal their identity: Jianhua Cang.

The manuscript was well received by both reviewers who commented on the importance of the work, the quality of the data and the clarity of the manuscript. However, they raised a number of issues that need to be addressed before the work is accepted for publication in eNeuro. These are summarized below.

1. Please respond to the comment concerning potential phase differences during the first and last period of the stimulus during different temporal frequencies.

2. Could you comment whether your data shed light on the relationship between adaptation suppression and stimulus selectivity?

3. Could you revise the layout of the figures, to make it consistent, as suggested by Reviewer 1?

4. When referencing relevant papers in the Introduction, be explicit which species were used in the studies they describe.

5. Please respond to the question about stimulus size and whether it affects sustained/transient nature of responses.

6. Please provide the definition of “adaptation” in the Introduction. Also, explain in the Introduction and in the abstract what you mean by the term “flexible”.

7. Please clarify whether you believe that responses to sudden stimulus appearance and responses to continuously present stimuli tap the same or different mechanisms.

8. Does orientation tuning you observed in mouse SC depend on topographic location of neurons within SC map?

9. Please address a few other points raised by reviewer 2, including figure legends, x-axis label in Fig 5c, text on lines 273, 358 and 479.

REVIEWER 1

ADVANCES THE FIELD

The authors studied surround suppression and temporal adaptation of superficial neurons in the mouse SC and reported several interesting observations.

COMMENTS TO THE AUTHORS

The authors performed extracellular recording of superficial SC neurons in awake mice. They studied their surround suppression and temporal adaptation and reported several interesting observations regarding the two properties and their interactions. The data were of high quality, data analysis appropriate, and writings clear. I only have a few very minor comments.

1. Stimulus temporal frequency was "usually" 4 Hz, but included a range. As such, when analyzing adaption, choosing the first 0.5 sec and last 0.5 sec could include different phases of the stimulus cycle. This could mis-represent the level of adaptation for cells that showed linear, phase locking, responses. I do not think this would change the main conclusions given the robust adaptation. But the authors may want to consider a slightly different methods, such as the first and last 2 cycles of visual stimulus.

2. Does surround suppression of adaptation correlate with the cells' direction/orientation selectivity? The authors should be able to answer this with their current data.

3. The figures' layout are not consistent. Some are organized by rows first (Fig. 1), some by column (Fig. 2), but Fig. 4 is a mix of both that should be corrected.

REVIEWER 2

ADVANCES THE FIELD

Exploring interactions between spatial and temporal processing is important to add to the SC literature.

COMMENTS TO THE AUTHORS

This paper studies mouse superior colliculus neurons with stimuli of different sizes and properties to explore surround suppression effects and how the initial transient response to stimulus onset evolves over time. The paper is generally clear, although there are places that need better attention as detailed below:

- In the introduction, sometimes the citations mix species, rendering the text potentially confusing and misleading (particularly to novices in the field). For example, around line 68, I felt that the general text was about mouse, but then there is a reference to Boehnke et al 2011 in monkey. The reference itself is fine, but I worry that the context of the text might mislead some readers. For example, the idea of SC superficial layers being "remarkably selective for image features" is not generally accepted in monkeys. So, if a reader reads the paragraph and misses that the focus in it was on mouse, and then this same reader sees citations with monkeys, then the reader might infer things that may not have been necessarily demonstrated yet in the monkey. A very simple remedy is to be very explicit in sentences about species-specific evidence such that citations are properly classified based on the species. So, I certainly do not intend to say that the Boehnke reference should be removed. Rather, keep it and others but with more clear delineation of how the evidence was obtained.

- line 234: were the full contrast sensitivity curves characterised at different sizes? e.g. Chen and Hafed 2018 looked at SC contrast sensitivity curves in different sizes. I think the curves changed slope with stimulus size, and there were also changes in how sustained activity (what you term adaptation) behaved. Small stimuli had higher sustained activity than large stimuli (I guess this means less adaptation in your context because the evoked responses in their case were generally of similar amplitude).

- line 260: related to the above, looking at Fig. 3a, the small stimulus has a slightly higher sustained response, as found in Chen and Hafed 2018 in monkeys (the monkey cells were generally more transient than sustained).

- line 273: missing "or" between "motion" and "direction"?

- having read the paper in full, I went back to the Boehnke et al 2011 citation mentioned in a comment above. In that study, I believe that adaptation was assessed by measuring the strength of the evoked response after repetitive presentation of a stimulus. However, in the present context, adaptation is meant the sustained response after the initial evoked visual burst. I think that the different paradigms (repetitive stimulus onset versus a continuously presented stimuli) can invoke different mechanisms. For example, your data show that surround suppression is stronger for the initial evoked response (Fig. 3) and almost absent for the sustained response. So, I think it would be best in the introduction (and discussion) to clarify what you mean by adaptation and what the other papers that you cited (like Boehnke et al) meant. I initially thought you will measure adaptation of the initial evoked response (by repeated onsets) in all of your experiments when I read the introduction (probably because I saw the Boehnke et al citation...). So, better be clear from the outset.

- the legends refer to figure parts as A, B, C...but the figure parts are labeled a, b, c...

- figure 5c (and also other figures): what does the x-axis label mean? "Direction re pref"? What does the "re" mean? I guess it means "direction relative to preferred direction" or something like that. Then, please write it in full. Also, please better write "preference" in full.

- line 318: I had a very hard time understanding the term "flexible" in the abstract, and I am still not sure even here this deep in the paper. Please clarify what is meant very early on....as early as in abstract, and then expand again on it in introduction.

- line 358: I don't understand this sentence. When the grating first appears, there's a transient response caused by luminance patterns of the gratings, and this evokes an initial transient response. How is the evoked response to the grating different from the evoked response to luminance transients?

- line 375: again, I think that there's a difference between measuring the initial response to a suddenly appearing stimulus with multiple repetitive onsets of this suddenly appearing stimulus and between measuring the response to a continuously presented stimulus at different times after its appearance. You measured the latter, but these other papers measured the former. Please clarify why and how you think they're tapping into the very same effects and mechanisms?

- Was there any dependence of the effects reported in this paper on topographic location of neurons within the SC map? For example, contrast sensitivity and spatial frequency tuning are quite different in upper and lower visual field representations in the monkey SC, and mouse SC has some dependencies of orientation tuning on topographic location.

- line 479: subsequent work in the monkey suggests that the links between visual responses and saccade latencies seems to be clearer for deeper than superficial neurons (e.g. Chen and Hafed, 2017; Chen et al., 2018).
